# Organized Neurogenic-Niche-Like Pinwheel Structures Discovered in Spinal Cord Tissue-Derived Neurospheres

**DOI:** 10.3389/fcell.2019.00334

**Published:** 2019-12-20

**Authors:** Francisco Javier Rodriguez-Jimenez, Eleonora Clemente, Victoria Moreno-Manzano, Slaven Erceg

**Affiliations:** ^1^Stem Cell Therapies in Neurodegenerative Diseases Laboratory, Principe Felipe Research Center (CIPF), Valencia, Spain; ^2^Neuronal Regeneration Laboratory, Principe Felipe Research Center (CIPF), Valencia, Spain; ^3^National Stem Cell Bank-Valencian Node, Platform for Proteomics, Genotyping and Cell Lines PRB3, Principe Felipe Research Center (CIPF), Valencia, Spain; ^4^Department of Tissue Cultures and Stem Cells, Institute of Experimental Medicine, Czech Academy of Sciences, Prague, Czechia

**Keywords:** pinwheels, astrocytes, ependymal cells, neurospheres, cilia

## Abstract

The neurogenic niche of the subventricular zone (SVZ) in adult brain tissue takes the form of a pinwheel-like cytoarchitectural structure, with mono-ciliated astrocytes displaying neural stem cell (NSC) characteristics present in the core surrounded by ciliated ependymal cells. For the first time, we have demonstrated the formation of similar pinwheel structures in spinal cord and SVZ tissue-derived neurospheres cultured *in vitro*. To investigate whether the organization and integrity of these pinwheel structures depends on the appropriate organization of ciliated astrocytes and ependymal cells, we modified neurosphere cell arrangements via the application of the methyltransferase inhibitor 5-aza-2′-deoxycytidine (5-aza-dc) or the antiviral drug ganciclovir (GCV) in transgenic mice expressing herpes simplex virus thymidine kinase from the GFAP promoter (GFAP-TK). Treatment of neurospheres with 5-aza-dc increased *FoxJ1* expression, a crucial factor for ciliogenesis, by reducing methylation of the FoxJ1 CpG island. 5-aza-dc also increased the expression of the astrocyte marker GFAP and caused aberrant accumulation of ciliated astrocytes. However, the ablation of dividing astrocytes within neurospheres by GCV treatment led to an increase in the accumulation of ciliated ependymal cells, as evidenced by the increased expression of the ependymal cell markers Vimentin or CD24. While 5-aza-dc and GCV treatment differentially affected cell arrangement, both compounds significantly diminished the number of pinwheel structures present in neurospheres. Thus, we suggest that the ratio of ciliated astrocytes to ependymal cells plays a crucial role in the correct formation of the pinwheel structures in spinal cord tissue-derived neurospheres *in vitro*.

## Introduction

Exploration of the subventricular zone (SVZ) of adult brain tissue has revealed the presence of a pinwheel structure specific to the adult neurogenic niche (Mirzadeh et al., 2008). The pinwheel core consists of mono-ciliated glial fibrillary acidic protein (GFAP)-positive astrocyte-like cells that possess properties of neural stem cells (NSCs) surrounded at the periphery by bi- or multi-ciliated ependymal cells. Previous studies identified GFAP-positive ciliated astrocytes located at the pinwheel core as dividing PH3/Ki67 double-positive cells with neurogenic potential (Mirzadeh et al., 2008; Cui et al., 2011). One of the primary functions of the motile cilia in cells of the central nervous system (CNS) is the circulation of cerebrospinal fluid (CSF). The cilia of astrocytes within the SVZ make contact with the ventricular fluid, and these mono-ciliated cells confer the pinwheel architecture to the ventricular surface in neurogenic regions of the adult brain and may regulate NSC behavior (Mirzadeh et al., 2008).

In spinal cord tissue, the ciliated ependymal stem-like cells that line the central canal possess limited self-renewal in the intact spinal cord (Alfaro-Cervello et al., 2012) and are bordered by a sub-ependymal layer (less elaborate than in the SVZ) containing small numbers of ciliated astrocytes, oligodendrocyte progenitors, and neurons (Hamilton et al., 2009; Alfaro-Cervello et al., 2012). Radial glial cells serve as primary progenitor cells in the CNS and can differentiate into ependymal stem-like cells and astrocytes during early postnatal periods (Jacquet et al., 2009). Interestingly, radial glial cells contain deuterosomes, which are associated with the early development of cilia, suggesting that cilia formation occurs during differentiation (Spassky et al., 2005). In addition to ependymal cells, cell subpopulations within the epithelium of the spinal cord central canal, such as dividing GFAP^+^ astrocytes (Alfaro-Cervello et al., 2012) also possess cilia.

In mammals, the FoxJ1 transcription factor regulates ciliogenesis and marks cells with motile cilia, including ependymal cells in the post-natal brain (Jacquet et al., 2009). Of the three FoxJ members identified (FoxJ1, 2, and 3), FoxJ2 and FoxJ3 are expressed during embryonic development, including in the brain (Landgren and Carlsson, 2004). FoxJ1-positive cells exhibit neurogenic potential *in vivo* and *in vitro*, with a subset of these cells considered as postnatal adult NSCs (Jacquet et al., 2009). Furthermore, the postnatal differentiation of ependymal stem cells and FoxJ1-positive astrocytes in the SVZ requires FoxJ1, while the absence of FoxJ1 associates with proliferation and differentiation defects resulting in severely abnormal olfactory bulb formation (Jacquet et al., 2009).

The *in vitro* culture of NSCs obtained from the SVZ and spinal cord leads to the formation of neurospheres (Reynolds and Rietze, 2005); however, we know little regarding the cellular organization and molecular mechanisms that determine the cell type proportion and distribution within neurospheres. In this study, we report for the first time that *in vitro* cultured spinal cord and SVZ neurospheres form pinwheel structures reminiscent of those present in the SVZ *in vivo*. We explored whether the proportion of ciliated astrocytes and ependymal cells influenced pinwheel formation and investigated the molecular mechanisms that control the final proportions of ciliated cells. We discovered that methylation of a CpG island located at −104 to +123 relative to the transcription start site of *FoxJ1* silences the FoxJ1 gene, and that forced demethylation by treatment with 5-azacytidine (5-aza-dc) rescues *FoxJ1* mRNA expression. In neurospheres derived from the transgenic mice expressing herpes simplex virus thymidine kinase from the GFAP promoter (GFAP-TK) treated with 5-aza-dc, we observed up-regulation of GFAP expression, indicative of a heightened number of astrocyte-like cells and the disruption of pinwheel structure. Alternatively, the presence of ganciclovir (GCV) causes the selective ablation of dividing astrocytes in the transgenic GFAP-TK mouse (Bush et al., 1998). Treatment leads to a decrease in GFAP expression and an increment in the levels of the Vimentin or CD24 ependymal markers in neurospheres obtained from GFAP-TK mouse *in vitro* (Imura et al., 2003) and, again, the disruption of pinwheel structure. Overall, modification of the distribution of ciliated astrocytes and ependymal cells significantly influences pinwheel arrangement and neurosphere formation of this organotypic-like culture *in vitro*, and this process may be critical to CNS development.

## Results

### The Presence of SVZ-Like Pinwheel Cytoarchitecture in Spinal Cord and SVZ-Derived Neurospheres

**FIGURE 1:**
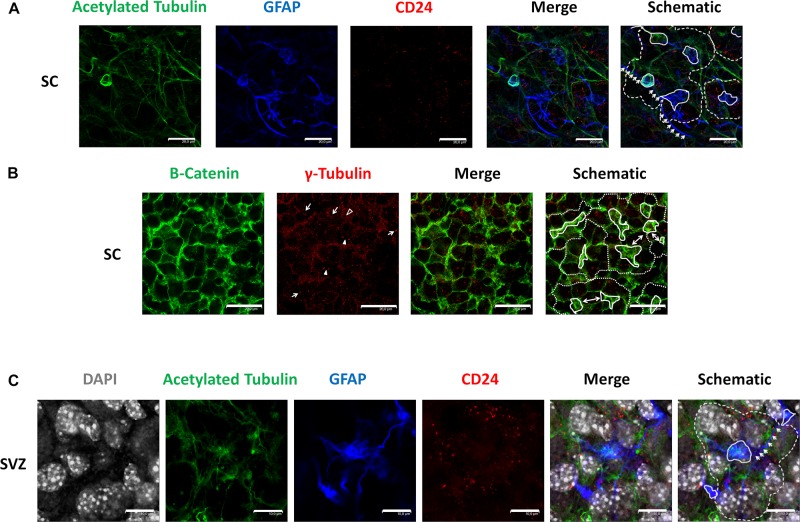
Identification of pinwheel structures in GFAP-TK mouse spinal cord and SVZ-derived neurospheres cultured *in vitro* **(A)** In spinal cord-derived (SC) neurospheres cultured *in vitro*, an acetylated tubulin (green) antibody was used to mark cilia, a GFAP (blue) antibody was used to mark astrocytes, a CD24 (red) antibody was used to mark ependymal cells. Central clusters of acetylated tubulin and GFAP detected are indicated by a continuous line, and the periphery of CD24-positive ependymal cells and the pinwheel structure is labeled by dashed lines (schematic). Lateral extensions of astrocytes are indicated by white arrows (schematic). **(B)** In spinal cord-derived (SC) neurospheres cultured *in vitro*, a β-catenin (green) antibody delineates the cell membrane, and an antibody against γ-tubulin (red) was used to detect single basal bodies marked by an empty arrowhead in small cells (as delineated by β-catenin). The γ-tubulin antibody also detected groups of basal bodies (marked by arrows) or double basal bodies (marked by arrowheads) in large cells (as delineated by β-catenin). Central clusters of γ-tubulin-positive small cells are indicated by a continuous line, and the periphery of the pinwheel structure is labeled by dashed lines (schematic). Single ependymal cells that are shared by two adjacent pinwheels are labeled by double-head arrows (i). **(C)** In SVZ-derived neurospheres cultured *in vitro*, an acetylated tubulin (green) antibody was used to mark cilia, a GFAP (blue) antibody was used to mark astrocytes, a CD24 (red) antibody was used to mark ependymal cells and DAPI was used to mark nuclei (gray). Central clusters of acetylated tubulin and GFAP detected are indicated by a continuous line, and the periphery of CD24-positive ependymal cells and the pinwheel structure is labeled by dashed lines (schematic). Lateral extensions of astrocytes are indicated by white arrows (schematic). The scale bars correspond to 20 **(A,B)** or 10 μm **(C)**.

We employed antibodies against acetylated tubulin to recognize cilia, GFAP as a marker of astrocytes, and CD24 as an ependymal stem cell marker (Liu et al., 2007) in GFAP-TK neurospheres (Figure 1A). Immunocytochemical evaluation of said markers revealed a distribution pattern similar to the pinwheel neurogenic-niche organization previously described in the SVZ of the adult brain (Mirzadeh et al., 2008). We observed the localization of GFAP (blue) and acetylated tubulin (green) at the pinwheel core (Figure 1A, continuous white lines in schematic) surrounded by staining for CD24 ependymal marker (Figure 1A, outlined by dashed lines in schematic). We also detected astrocyte-like extensions that connected adjacent core centers in neurospheres (Figure 1A, indicated by white arrows in the schematic) similar to those described in the SVZ (Mirzadeh et al., 2008).

We employed a β-catenin antibody to delineate cell borders and explored the protein distribution of γ-tubulin in GFAP-TK neurospheres cultured *in vitro* using an antibody that recognizes γ-tubulin in microtubule-organizing centers (MTOCs), centrosomes (Oakley, 1992), and basal bodies (Mirzadeh et al., 2008; Figure 1B). By immunocytochemical evaluation of GFAP-TK spinal cord-derived neurospheres, we encountered γ-tubulin and β-catenin distribution patterns similar to the pinwheel neurogenic-niche organization of the SVZ (Figure 1B, outlined by dashed lines in the schematic). When studying γ-tubulin patterning, we encountered clusters of small basal bodies (marked by arrows) or double basal bodies (marked by filled arrowheads) in large ependymal cells (delineated by β-catenin staining) (Figure 1B). We also observed regions of small cells delineated by β-catenin (Figure 1B, indicated by continuous white lines in schematic) containing a single basal body detected by γ-tubulin (Figure 1B, an example marked by empty arrowhead), similar to structures usually positioned at the pinwheel structure core identified as astrocytes in the SVZ (Mirzadeh et al., 2008). We also note that, as observed in the SVZ (Mirzadeh et al., 2008), some single ependymal cells helped to form two adjacent pinwheels in GFAP-TK spinal cord-derived neurospheres (Figure 1B, labeled by double-headed arrows in schematic). We also show, for the first time (Figure 1C), that neurospheres obtained from adult SVZ present a similar organization to that observed in the SVZ and GFAP-TK spinal cord-derived neurospheres (Figure 1A). Nuclei of large ependymal cells and small astrocytes are labeled by DAPI (gray). Nuclei of astrocytes (blue) seem to be present in a deeper layer (Figure 1C, outlined by continuous white lines in schematic), suggesting a stratification of neurospheres in a manner similar to that described for the SVZ. We also detected astrocyte extensions that connect adjacent core centers (Figure 1C, indicated by white arrows in schematic) similar to those described in the SVZ (Mirzadeh et al., 2008) and GFAP-TK spinal cord-derived neurospheres (Figure 1A).

We next sought to investigate the role of the ciliated cells that make up the SVZ-like pinwheel formed by GFAP-TK spinal cord-derived neurospheres by first targeting the expression of FoxJ1 in ciliated cells via epigenetic modulation.

### DNA Methylation of the FoxJ1 CpG Island Regulates Gene Expression in Spinal Cord-Derived Neurospheres

**FIGURE 2:**
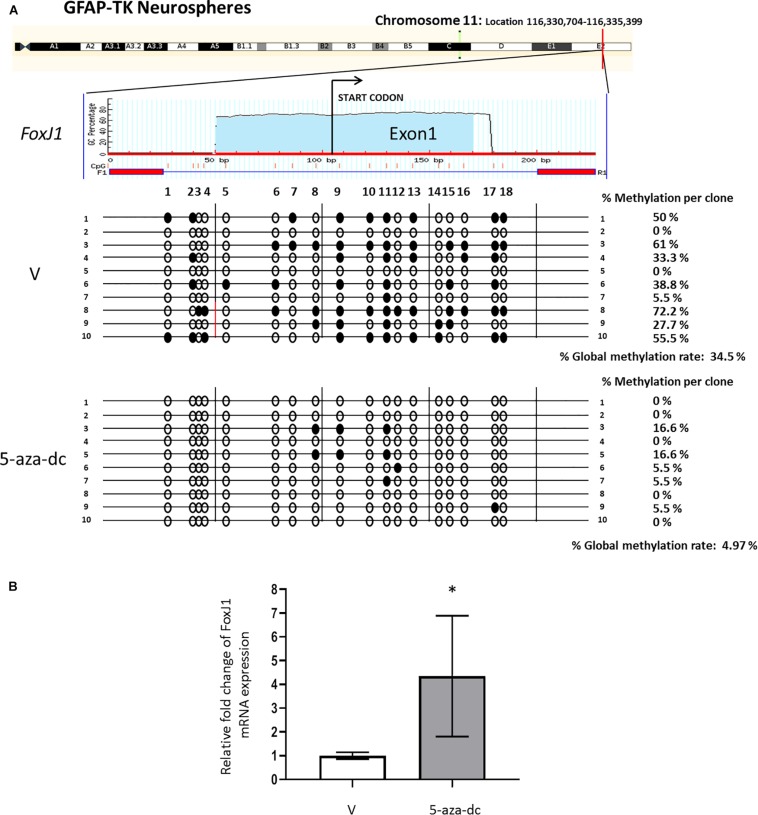
Identification and analysis of FoxJ1 CpG island GFAP-TK mouse spinal cord-derived neurospheres cultured *in vitro*. **(A)** GFAP-TK neurospheres cultured for 2 weeks were treated with vehicle (DMSO [V]) or 5-aza-dc for 48 h. A schematic of the FoxJ1 CpG island is shown at the top for reference with the transcription start point indicated by an arrow. Each row represents an individually cloned and sequenced allele following sodium bisulfite DNA modification. Ten clones were analyzed per condition. The grade of the individual CpG island methylation for each clone and the global methylation per condition is indicated as a percentage of methylation. Vertical lines beneath the horizontal line indicate the distributions and densities of CpG sites. CpG sites are marked as circles to accurately reflect the CpG density of the region, with empty circles for unmethylated CpG sites and filled circles for methylated CpG sites. **(B)** FoxJ1 relative mRNA expression was determined by real-time PCR, and the values normalized to GAPDH. Values are represented as mean ± SD, *n* = 6. ^∗^*p* < 0.05 determined by Student’s *t*-test was considered statistically significant.

We first analyzed the promoter region and first exon of the *FoxJ1* gene [chromosome 11: Location 116,330,704-116,335,399 (reverse strand)] to discover a possible CpG island using the MethPrimer software. We detected a CpG island at the 5′upstream region of *FoxJ1* (−104 to +123 relative to the transcription start site) and designed primers (amplified a 227 bp PCR product that includes 18 CpG sites) for bisulfite analysis. Methylation status analysis of the described region in at least ten plasmid clones 2 weeks after spinal cord extraction revealed 34.5% methylated CpG sites in neurospheres treated with vehicle [DMSO (V), in all cases] for 48 h. Treatment with the 5-aza-dc methyltransferase inhibitor (10 μM) for 48 h reversed promoter hypermethylation (4.97%) (Figure 2A) and significantly increased the fold change of *FoxJ1* gene expression in GFAP-TK neurospheres when compared to basal levels (DMSO 1 ± 0.14 vs. 5-aza-dc 4.34 ± 2.54; *p* < 0.05) (Figure 2B).

### 5-aza-dc and GCV Treatment Alter Astrocyte and Ependymal Cell Markers

**FIGURE 3:**
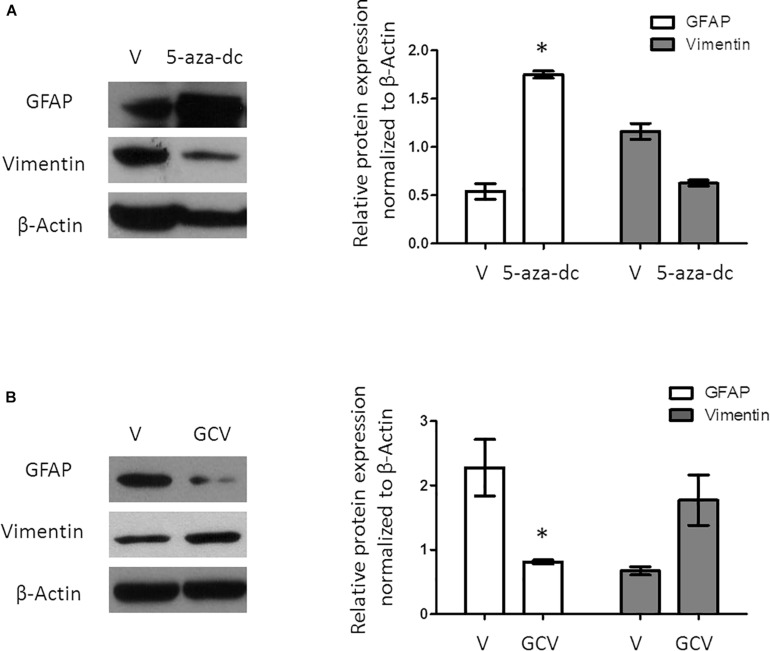
Protein expression analysis of GFAP and Vimentin in GFAP-TK mouse spinal cord-derived neurospheres cultured *in vitro*. **(A)** GFAP-TK neurospheres cultured for 2 weeks were treated with vehicle (DMSO [V]) or 5-aza-dc (48 h). Analysis of protein expression (a) by Western blotting used specific antibodies against GFAP and Vimentin. Densitometry analysis of proteins was performed using the ImageJ software. Values were normalized to β-Actin in three independent experiments and results represented in the graphics. **(B)** GFAP-TK neurospheres cultured for 2 weeks were treated with vehicle (DMSO [V]) or GCV (24 h). Analysis of protein expression was quantified by Western blotting (a), and the corresponding quantification by densitometry is shown. ^∗^*p* < 0.05, determined by a Mann Whitney *U* test were considered statistically significant.

A previous study revealed that a population of GFAP-positive cells in the SVZ requires the expression/activity of the FoxJ1 transcription factor. As 5-aza-dc increases the mRNA expression of *FoxJ1*, we evaluated the expression of the astrocyte cell marker GFAP after treatment with 5-aza-dc for 48 h showing a significantly increases of protein expression (DMSO 0.5375 ± 0.16 vs. 5-aza-dc 1.74 ± 0.074; *p* < 0.05), but slightly decreases the expression of the ependymal cell marker Vimentin (DMSO 1.15 ± 0.14 vs. 5-aza-dc 0.62 ± 0.052; ns). Overall, our protein analysis suggests an enrichment in ciliated astrocytes at the expense of ciliated ependymal cells (Figure 3A).

### Selective Ablation of Ciliated Astrocytes Leads to an Enrichment of Ciliated Ependymal Cells

Ganciclovir treatment selectively eliminates dividing GFAP-positive cells in the spinal cord as well as in spinal cord-derived neurospheres (Bush et al., 1998; Imura et al., 2003). Treatment of GFAP-TK neurospheres with GCV for 24 h significantly decreased the protein expression of GFAP (DMSO 2.27 ± 0.88 vs. GCV 0.67 ± 0.10; *p* < 0.05) but slightly increased Vimentin (DMSO 0.81 ± 0.05 vs. GCV 1.77 ± 0.67; ns) suggesting the enrichment in ciliated ependymal cells to the detriment of ciliated astrocytes (Figure 3B).

### 5-aza-dc and GCV Treatment Alter the Cellular Distribution and Organization of Ciliated GFAP-Positive and CD24-Positive Cells in GFAP-TK Spinal Cord-Derived Neurospheres

**FIGURE 4:**
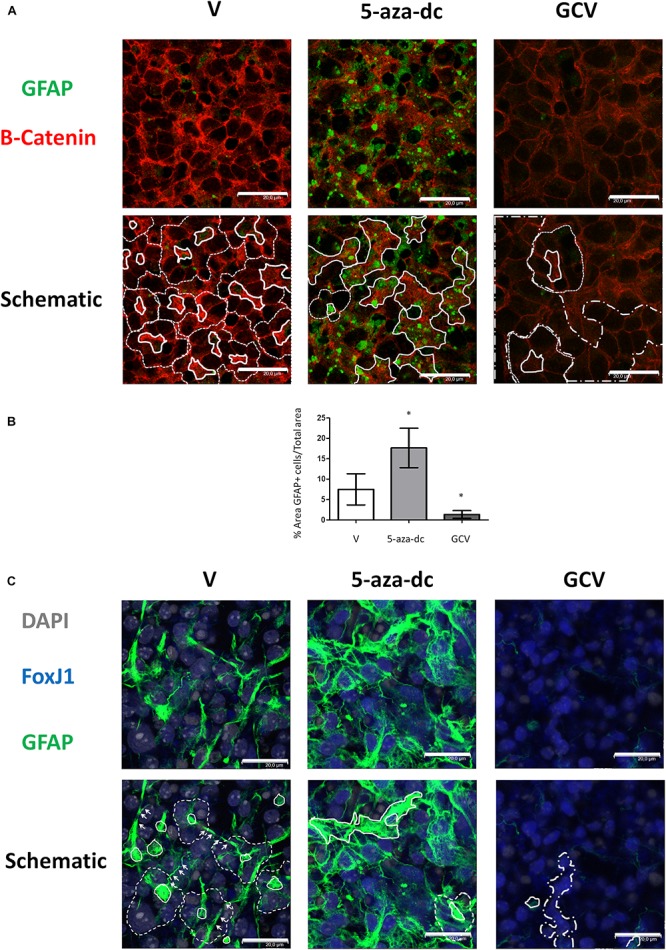
GFAP astrocyte marker expression in GFAP-TK mouse spinal cord-derived neurospheres cultured *in vitro* and treated with 5-aza-dc or GCV. **(A)** GFAP-TK neurospheres were treated with vehicle (DMSO [V]), 5-aza-dc, or GCV. The GFAP signal was obtained by using a rabbit polyclonal anti-GFAP antibody that marks astrocytes (green) while the β-catenin signal delineates cell borders (red). Pinwheel structures identified are marked with dashed lines indicating the periphery of ependymal cells and a solid white line indicating the core (schematic). In 5-aza-dc treatment, the continuous lines mark the core of pinwheel and aberrant accumulations of GFAP-positive cells (schematic). In GCV treatment, dashed and dotted lines mark regions of accumulated large cells (schematic). Scale bars, 20 μM. **(B)** Percentage of GFAP positive areas in twenty regions over multiple experiments (mean ± SD) were calculated for each condition using ImageJ software and compared to total area. ^∗^*p* < 0.05 determined by Student’s *t*-test was considered statistically significant. **(C)** The GFAP signal obtained by using a chicken polyclonal anti-GFAP antibody marks astrocytes (green), the FoxJ1 signal (blue) and the nuclear marker DAPI (gray). Lateral extensions of astrocyte connecting with astrocytes from adjacent cores are indicated by arrows. Scale bars, 20 μM.

In the SVZ, GFAP-positive astrocytic cells are present as small cells located at the pinwheel core (Mirzadeh et al., 2008). We analyzed the distribution of GFAP-positive cells in GFAP-TK spinal cord-derived neurospheres cultured for 2 weeks *in vitro* and after treatment with vehicle [DMSO (V)], 5-aza-dc, or GCV using a rabbit polyclonal anti-GFAP antibody (Figure 4A). We employed a β-catenin antibody to delineate cell borders. We identified the pinwheel structures with GFAP-positive cells at the core (Figure 4A, delineated by solid white line in schematic) surrounded by large GFAP-negative cells in vehicle-treated samples (Figure 4A, delineated by dashed white line in schematic). However, we observed the accumulation of clusters of small GFAP-positive cells (Figure 4A, marked by solid white line in schematic) and the disruption of the pinwheel cytoarchitecture in 5-aza-dc treated samples. GCV treatment favored the accumulation of large GFAP-negative cells identified as ependymal cells (Figure 4A, marked by dashed-dotted lines in schematic). Despite the disruption to the pinwheel cytoarchitecture by 5-aza-dc and GCV treatment, we still detected the presence of a few remaining pinwheels (Figure 4A, marked by dashed line in schematic). Overall, we observed a significant increase in the area representing GFAP-positive cells in GFAP-TK spinal cord-derived neurospheres treated with 5-aza-dc (DMSO 7.5 ± 3.8 vs. 5- aza-dc 17.3 ± 5.86; *p* < 0.05) and a significant decrease in those treated with GCV (DMSO 7.5 ± 3.8 vs. GCV 1.33 ± 0.9; *p* < 0.05). The graph shows the overall results obtained from twenty images for each condition (Figure 4B). We also obtained comparable results using a chicken polyclonal anti-GFAP antibody (Figure 4C). We mainly observed FoxJ1 in large nuclei (DAPI stained) of positive cells surrounding GFAP positive cells, in line with the results obtained *in vivo* (Redmond et al., 2019). Lateral extensions of astrocyte connecting with neighboring astrocytes from adjacent cores in neurospheres are marked by white arrows (Figure 4C).

**FIGURE 5:**
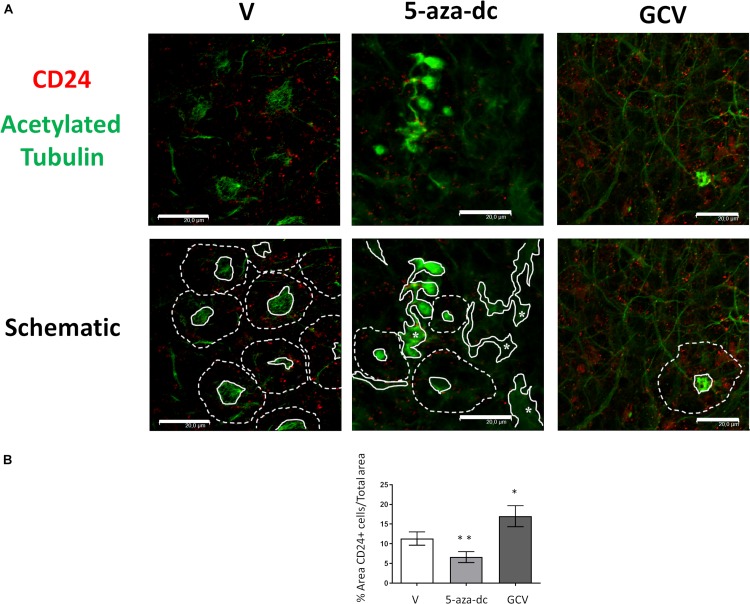
CD24 ependymal cell marker expression in GFAP-TK mouse spinal cord-derived neurospheres cultured *in vitro* and treated with 5-aza-dc or GCV. **(A)** GFAP-TK neurospheres were treated with vehicle [DMSO (V)], 5-aza-dc, or GCV. CD24 signal marks ependymal cells (red) while acetylated tubulin signal marks cilia (green). Pinwheel structures detected are marked with dashed lines indicating the periphery of ependymal cells and a solid white line indicating the core (schematic). Continue lines containing an asterisk mark the aberrant accumulation of acetylated tubulin. Scale bars, 20 μM. **(B)** Percentage of CD24 positive areas in 20 regions over multiple experiments (mean ± SD) were calculated for each condition using ImageJ software and compared to the total area. ^∗^*p* < 0.05, ^∗∗^*p* < 0.01 determined by Student’s *t*-test were considered statistically significant.

We next analyzed the distribution of the ependymal marker CD24 (red) and acetylated tubulin (green) in GFAP-TK neurospheres treated with vehicle [DMSO (V)], 5-aza-dc, or GCV (Figure 5A). In DMSO-treated GFAP-TK spinal cord-derived neurospheres, we observed CD24 staining indicative of ependymal cells in those cells surrounding the center of the pinwheel (Figure 5A, marked by dashed white line in schematic) and rarely at the core (Figure 5A, marked by solid white line in schematic), similar to the SVZ (Mirzadeh et al., 2008). We found a similar CD24 staining pattern in GFAP-TK neurospheres treated with 5-aza-dc, where even given the disruption of the pinwheel cytoarchitecture, these structures remained present (Figure 5A). We observed abnormal accumulations of acetylated tubulin surrounded by CD24 positive cells in GFAP-TK neurospheres treated with 5-aza-dc (Figure 5A, indicated by asterisks and solid lines in schematic). In GFAP-TK neurospheres treated with GCV, we detected larger areas of CD24 positive cells with little presence of acetylated tubulin accumulation that corresponds to pinwheel cores (Figure 5A). Even given the disruption of the pinwheel cytoarchitecture, we still detected these structures (Figure 5A; schematic).

Overall, treatment with 5-aza-dc significantly decreased the percentage of CD24 positive area in cells treated compared to vehicle (DSMO 11.34 ± 1.68 vs. 5-aza-dc 6.623 ± 1.38; *p* < 0.01), while GCV caused a significant increase in the percentage of CD24 positive area of cells treated compared to vehicle (DSMO 11.34 ± 1.68 vs. GCV 17.013 ± 2.63; *p* < 0.05) (Figure 5B).

**FIGURE 6:**
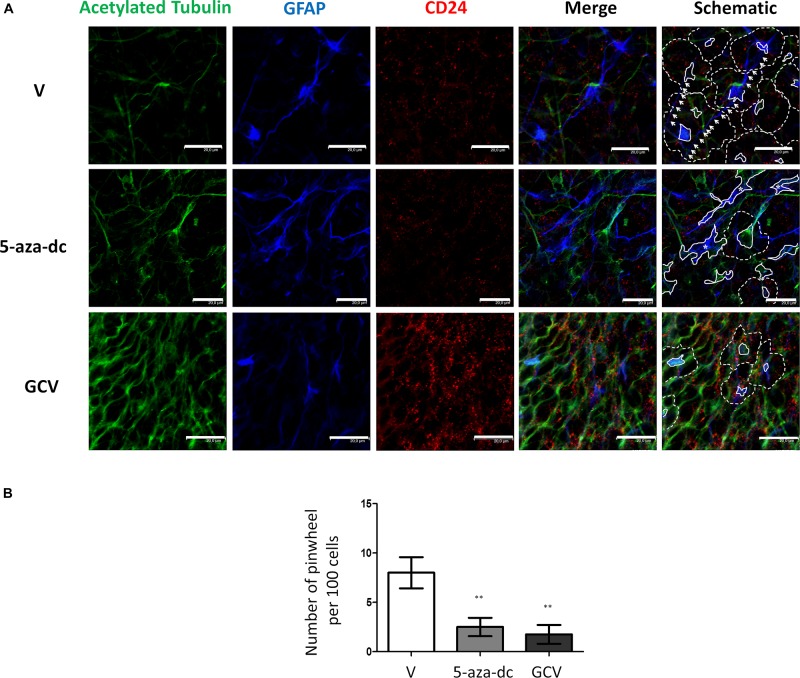
GFAP astrocyte marker and CD24 ependymal cell expression in GFAP-TK mouse spinal cord-derived neurospheres cultured *in vitro* and treated with 5-aza-dc or GCV. **(A)** Cells treated with vehicle (DMSO [V]), 5-aza-dc, or GCV. Acetylated tubulin (green) antibody was used to mark cilia, a CD24 (red) antibody was used to mark ependymal cells, and a GFAP (blue) antibody was used to mark astrocytes, and all are shown in the merge. Central clusters are marked by a solid white line, and cores with aberrant accumulations of acetylated tubulin and GFAP detected after 5-aza-dc treatment are marked by asterisks and by a solid white line (schematic). CD24-positive ependymal cells and the pinwheel periphery are labeled by dashed lines (schematic). Astrocyte extensions that connect adjacent cores were labeled by white arrows (schematic). **(B)** Quantification of pinwheel structures in GFAP-TK mouse spinal cord-derived neurospheres cultured *in vitro*. The graphic displays the number of pinwheels counted per 100 total cells and from twenty pictures per condition after immunocytochemical evaluation. ^∗∗^*p* < 0.01 determined by Student’s *t*-test was considered statistically significant.

Finally, we evaluated a combination of antibodies for the clear identification of the pinwheel structures under different conditions: we used an antibody against acetylated tubulin and GFAP to identify the cores and an antibody against CD24 to mark the surface of surrounding ependymal cells (Figure 6A). We treated cells with vehicle [DMSO (V)], 5-aza-dc, or GCV. After vehicle [DMSO (V)] treatment, we continued to observe a well-organized distribution of pinwheels formed by cores labeled by acetylated tubulin and GFAP (Figure 6A, marked by solid lines in schematic) and surrounded by CD24-positive cells (Figure 6A, outlined by dotted lines in schematic). As described in SVZ tissue, we also observed lateral extensions of astrocyte connecting with astrocytes from adjacent cores in neurospheres (Figure 6A, marked by white arrows in schematic). As previously described, treatment with 5-aza-dc caused abnormal accumulations of GFAP and acetylated tubulin that indicate the cores (Figure 6A, indicated by asterisks and solid lines in schematic). In GFAP-TK neurospheres treated with GCV, we observed larger areas positive for CD24 and a lack of GFAP and acetylated tubulin signal (Figure 6A).

Following vehicle [DMSO (V)] treatment of GFAP-TK spinal cord-derived neurospheres, we also encountered similar γ-tubulin and β-catenin distribution patterns for the described pinwheel neurogenic-like niche (Supplementary Figure S1A, demarcated by dashed white lines in schematic). Interestingly, we observed that pinwheels became distributed in row-like formations (Supplementary Figure S1A, marked by numbers in schematic). However, the treatment of GFAP-TK spinal cord-derived neurospheres with 5-aza-dc led to the disruption of the pinwheel-like structure and the accumulation of small γ-tubulin-positive cells (Supplementary Figure S1A, delimited by continuous line and marked by asterisks in schematic). In neurospheres treated with GCV, we observed large cells that may correspond to ependymal cells residing in close vicinity, while we noted a general lack of cores of small cells (Supplementary Figure S1A). GCV treatment also largely disrupted the pinwheel structure and promoted the accumulation of γ-tubulin aggregates (Supplementary Figure S1A; marked by white dots in schematic) within larger cells as delineated by β-catenin. The accumulation of γ-tubulin due to abnormal distribution of γ-tubulin protein at the apical surface has been previously described (Jacquet et al., 2009).

Moreover, we explored the distribution of basal bodies and cilia, observing a well-organized distribution of cores labeled by acetylated tubulin and γ-tubulin after vehicle [DMSO (V)] treatment (Supplementary Figure S1B) with cores distributed into rows (Supplementary Figure S1B, as indicated by dotted arrows, schematic). However, after 5-aza-dc treatment, we frequently observed large accumulations of both markers (Supplementary Figure S1B; indicated by asterisks and solid white line in schematic). Treatment with GCV caused not only a large accumulation of γ-tubulin labeling as reported above but also acetylated tubulin labeling (Supplementary Figure S1B, marked by solid lines with white dots in schematic), thereby confirming alterations to the cilia.

Finally, we determined the number of pinwheel-like structures per 100 cells in at least 20 pictographs per condition by immunocytochemistry for GFAP-TK spinal cord-derived neurospheres. We discovered a lower number of pinwheel-like structures in GFAP-TK neurospheres treated with 5-aza-dc (DMSO 8 ± 1.58 vs. 5-aza-dc 2.5 ± 0.925; *p* < 0.01) or GCV (DMSO 8 ± 1.58 vs. GCV 1.75 ± 0.95; *p* < 0.01) when compared to vehicle [DMSO (V)] treated cells (Figure 6B).

## Discussion

The neurogenic capacity of the mouse SVZ depends on the capability of ependymal cells to assemble into pinwheels; unique structures that constitute real regenerative units (Paez-Gonzalez et al., 2011; Gonzalez-Cano et al., 2016). In neurogenic regions of the SVZ in the mouse adult brain, the cytoarchitecture of the pinwheel unit constitutes a core of ciliated astrocytes surrounded by ciliated ependymal cells (Mirzadeh et al., 2008). In the spinal cord, ependymal cells with stem cell properties (Barnabe-Heider et al., 2010) line the central canal with bordering astrocytes. Thus, since ependymal cells and astrocytes do not form pinwheel in the spinal cord, one would not expect these structures in spinal cord-derived neurospheres *in vitro*, unless the artificial proliferative conditions *in vitro* mimic a microenvironment similar to the neurogenic-like niche *in vivo*. Here we report that neurospheres from spinal cord cultured *in vitro* contain pinwheel-like structures similar to the neurogenic niche in the adult SVZ. This neurosphere cytoarchitecture could confer neurogenic niche-like properties that would represent an exciting source of transplantable neurogenic cells.

FoxJ1 is the master regulator of ciliogenesis and governs a specific motile ciliogenic program in cells. FoxJ1 is necessary for the differentiation of radial glial cells into a subset of astrocytes and ependymal cells in the postnatal brain (Jacquet et al., 2009). Ciliated astrocyte cells attribute unique pinwheel architecture to the ventricular surface in neurogenic regions of the adult brain (Mirzadeh et al., 2008). Some studies have shown a link between FoxJ1 and the astrocytic marker GFAP; in fact, the maintenance of the population of GFAP-positive cells in SVZ requires FoxJ1 (Jacquet et al., 2009). Moreover, disruption of the FoxJ1/Ank-3 pathway *in vivo* results in a dramatic reduction of neuroblasts and ependymal niche organization in the brain (Paez-Gonzalez et al., 2011). Thus, we explored whether an alteration to the distribution of comprising cells could influence the proper formation of the niche. The regulation of *FoxJ1* in ciliated ependymal cells and astrocytes from the spinal cord, as well as its possible contribution to neurosphere organization *in vitro*, remains mostly unexplored. Here we show that DNA methylation epigenetically regulates *FoxJ1* expression; treatment of neurospheres with the DNA methylation inhibitor (5-aza-dc) reduced the overall level of CpG island methylation and induced *FoxJ1* expression. The presence of FoxJ1 is necessary for the appropriate expression of proteins involved in the generation of motile cilia, such as γ-tubulin (Jacquet et al., 2009; Didon et al., 2013).

Treatment of GFAP-TK neurospheres from the spinal cord with 5-aza-dc increases GFAP and decreases Vimentin and CD24 protein expression, while GCV increases Vimentin and CD24 and decreases GFAP expression. Treatment with 5-aza-dc causes the enrichment of the astrocyte marker GFAP in GFAP-TK neurospheres regardless of FoxJ1 expression, possibly via the epigenetic regulation of GFAP (Restrepo et al., 2011), while GCV favors an increase in the ependymal cell population. Analysis of the distribution of expression of acetylated tubulin (a cilia-related protein), GFAP (astrocyte marker), and CD24 (ependymal cell marker) in GFAP-TK neurospheres revealed that treatment with 5-aza-dc causes the accumulation of acetylated tubulin/GFAP double-positive small cells in comparison to GFAP-TK neurospheres treated with vehicle [DMSO (V)]. In contrast, treatment with GCV causes areas of accumulation of large cells and an increased percentage of CD24-positive cells. After GCV treatment, we frequently observed that γ-tubulin and acetylated tubulin were not distributed as before nor docked at the apical surface of cells and instead formed aberrant aggregates as shown for FoxJ1-null cells (Jacquet et al., 2009). This result may indicate that killing dividing astrocytes by GCV may influence the correct distribution of γ-tubulin and acetylated tubulin and, therefore, cilia formation in cells forming neurospheres.

After identification and enumeration of pinwheels present in neurospheres from spinal cord cultured *in vitro*, we observed a significant decrease in pinwheel number following treatment with 5-aza-dc or GCV compared to vehicle. Thereby, modification of the internal distribution of astrocytes and ependymal cells by these drugs affects the proper assembly of the pinwheel cytoarchitecture in spinal cord neurospheres cultured *in vitro*. Our study is the first to describe report that pinwheel structures located in the SVZ of the brain *in vivo* also occur in SVZ and spinal cord-derived neurospheres cultured *in vitro.* However, we note the requirement for additional studies to determine the apical surface position of γ-tubulin, cell polarity, and the orientation of cells in the neurosphere to assess whether these structures faithfully mimic the *in vivo* cellular distribution and tissue structures of SVZ pinwheels. The results obtained in our study suggest that cells of the neuronal lineage may possess an intrinsic memory and capability to self-organize in a similar manner to the adult tissue, behaving like an organotypic-like culture. Therefore, we believe that the *in vitro* culture of these cells will be of significant use to high-throughput genomic and proteomic approaches aiming to assess biological function, as well as to drug evaluation screens to study neurogenic tissue formation. This organotypic-like culture resembles the organization of the neurogenic niche of the SVZ in the adult brain may offer an opportunity to study mechanisms of neurogenesis under normal or pathological conditions. In addition, the described *in vitro* organized microenvironmental niche could influence cell behavior mimicking SVZ development, thereby presenting obvious interest to the regenerative medicine field.

In conclusion, the analysis presented here suggests that ciliated astrocytes and ependymal cells are necessary for the faithful assembly of the pinwheel structures *in vitro* in a process that may also be pertinent for the *in vivo* formation neurogenic niches of the CNS.

## Materials and Methods

### Wild Type and Transgenic Animals

Transgenic mice generated as previously described were used for the selective ablation of dividing astrocytes. The GFAP-TK mouse is a transgenic model in which thymidine kinase (TK) from the herpes simplex virus (HSV) is driven by the mouse GFAP promoter. This permits selective ablation of dividing GFAP-TK-expressing astrocytes following administration of the antiviral drug GCV, both *in vitro* and *in vivo*. Adult female GFAP-TK transgenic mice and wild type (WT) mice were obtained by mating heterozygous females of GFAP-TK line 7.1 with wild type C57/BL6 males.

#### GFAP-TK Neurosphere Isolation and Treatment

Neurospheres were obtained from the spinal cord of GFAP-TK transgenic mice (Bush et al., 1998) at 3 months of age, as previously described (Moreno-Manzano et al., 2009). Briefly, once the overlying meninges and blood vessels were removed, the dissected spinal cord tissue was placed in fresh washing medium (DMEM/F12 supplemented with 100 U/ml penicillin, 100 μg/ml streptomycin, 5 mM HEPES buffer, 0.125% NaHCO3, 0.09% glucose) and cut into 1-mm^3^ pieces. Under sterile conditions, the tissue was homogenized; first, by incubating in washing medium for 10 min at 37°C, then by disaggregating down to the cellular level in growth medium and passing 20 times through a thin silicon-glass pipette. GFAP-TK neurospheres were formed in growth medium in low-attachment plates [DMEM/F12 supplemented with 100 U/ml penicillin, 100 μg/ml streptomycin, 2 mM L-glutamine, 5 mM HEPES buffer, 0.125% NaHCO_3_, 0.6% glucose, 0.025 mg/ml insulin, 80 μg/ml apotransferrin, 16 nM progesterone, 60 μM putrescine, 24 nM sodium selenite, 4 μg/ml bovine serum albumin (BSA), 0.7 U/ml heparin, 20 ng/ml epidermal growth factor (EGF) and 10 ng/ml basic fibroblast growth factor (FGF)]. GFAP-TK neurospheres were isolated and cultured for 2 weeks. Next, GFAP-TK neurospheres were treated with either vehicle (dimethyl sulfoxide, DMSO), the methyltransferase inhibitor 5-aza-dc dissolved in DMSO for 48 h (10 μM; Sigma Chemical, Mo., United States), or GCV in DMSO for 24 h (3 μM; Sigma Chemical, Mo., United States). Adult brains were cut into coronal sections using a tissue chopper, and the sections were collected in 6-cm Petri dishes filled with 3 ml of complete growth medium for neurospheres. We dissected the lateral walls of the lateral ventricles under a dissecting microscope and keep the dissected tissue in tubes, each containing 5 ml of growth medium. Tissue was homogenized like spinal cord tissue and neurospheres grown for 2 weeks before using.

### DNA Extraction, Purification, Bisulfite Modification, and Sequencing

CpG island methylation analysis of the *FoxJ1* gene in GFAP-TK neurospheres was performed by bisulfite treatment and sequencing (BSP). Genomic DNA was extracted using a DNeasy Blood and tissue kit (QIAGEN). Genomic DNA (400 ng) was treated with sodium bisulfite using the EZ DNA methylation kit (Zymo Research). Bisulfite-treated DNA was amplified with primers designed to amplify both methylated and unmethylated DNA (BSP) to cover the CpG-rich dinucleotide region of the *FoxJ1* gene. Primers were designed using MethPrimer software. Particularly, from the location: 116,330,704-116,335,399 (Chromosome 11). BSP *FoxJ1* sense primer (5′- ATTGTGTAATTGGAGTTTGGGTTAT -3′) and BSP *FoxJ1* antisense primer (5′- TTCCTACAACCATTACAAACTAATCAA -3′) were used. PCR products were then sub-cloned into the pGEMT vector (Promega), and DNA was isolated from at least ten clones per condition. Finally, samples were sequenced in both directions using the BigDye Terminator cycle sequencing kit (Applied Biosystems) and ABI 310 automated sequencer (the average methylation levels per individual clones are represented in Figure 2).

### Total RNA Extraction and Real-Time Polymerase Chain Reaction

Total RNA was obtained using the NucleoSpin RNA/Prot kit (Macherey Nagel, Germany) and reverse-transcribed using TaqMan reverse transcription reagents (Applied Biosystems) following the manufacturer’s instructions. As a template, 40 ng cDNA from target and housekeeping genes were independently prepared for each TaqMan reaction. Each reaction was performed in duplicate from three independent experiments. The MGB assay on demand TaqMan probe (Applied Biosystems) for *FoxJ1* (Mm01267279_m1) was used, and the results were normalized to glyceraldehyde-3-phosphate dehydrogenase (*Gapdh*) (Mm99999915_g1) used as a housekeeping gene. The comparative threshold cycle (CT) method was used to calculate the relative expression (Livak and Schmittgen, 2001).

### Western Blotting Analysis

Cells were collected and proteins extracted using Lysis Buffer - 50 mM Tris–HCl, pH 7.5, 150 mM NaCl, 0.02% NaN3, 0.1 SDS, 1% NP40, 1 mM EDTA, 2 μg/ml leupeptin, 2 μg/ml aprotinin, 1 mM PMSF, 1 x Protease Inhibitor Cocktail (Roche Diagnostics, IN, United States). Equal amounts of protein extracts (50 μg) were loaded onto a 10% SDS-polyacrylamide gel and resolved by standard SDS-PAGE. Proteins were then electrophoretically transferred onto PVDF membranes. Membranes were blocked with 5% skimmed milk in PBST for 60 min and tested overnight with specific antibodies at dilution 1:1000 against, mouse monoclonal glial fibrillary acidic protein (GFAP; M0761; Dako, Denmark); mouse monoclonal Vimentin (Abcam, ab8978) and mouse monoclonal ß-actin at dilution 1:5000 (A5441; Sigma Chemical, MO, United States) which was used as loading control. Subsequently, membranes were incubated with rabbit anti-mouse or goat anti-rabbit horseradish peroxidase-conjugated secondary antibody (1:5000) (Sigma Chemical, St. Louis, MO, United States). Blots were visualized by the ECL detection system (Amersham, United Kingdom). Results were quantified by densitometry using ImageJ Software.

### Immunocytochemistry

GFAP-TK neurospheres were grown for 6 h, as previously described (Rodriguez-Jimenez et al., 2015) to allow them to adhere to a poly-L-lysine-coated cover slide (Sigma-Aldrich; P4707). The medium employed here differs from the neurosphere growth medium described above by the addition of heparin and the removal of the mitogenic factors EGF and FGF and the replacement of BSA by 2% fetal bovine serum (FBS). Cells were post-fixed with 4% paraformaldehyde (PFA) at room temperature for 15 min. After permeabilization of cell membranes with 0.1% Triton X-100, samples were blocked with 10% FBS. Incubation with primary antibodies was performed overnight (1:200) at 4°C; mouse monoclonal FoxJ1 (Invitrogen, 4-9965-82), mouse monoclonal glial fibrillary acidic protein (GFAP; M0761; Dako, Denmark), chicken polyclonal GFAP (Thermofisher, PA1-10004), rat monoclonal CD24 (BD Biosciences; 557436), mouse monoclonal acetylated tubulin (Sigma Aldrich; T6793), rabbit polyclonal γ-tubulin (Sigma Aldrich; T5192), mouse monoclonal ß-Catenin (Transduction Laboratories; 610153 BD), and rabbit polyclonal antibody ß-Catenin [Cell Signaling Technology; 9562). After a wash step, secondary antibodies (Oregon green 488 goat anti-mouse IgG; Alexa Fluor 555 goat anti-rat IgG or Alexa Fluor 647 mouse anti-rabbit (1:400; Life Technology, United States)], were incubated with the sections for 1 h at room temperature. Signals were visualized by confocal microscopy (Leica). Quantification of GFAP/CD24 positive areas was performed using ImageJ software; the area of positive signal is expressed as a percentage of the total area from twenty pictures.

### Statistical Analysis

Statistical analyses were performed using the software GraphPad Prism 5. Two-tailed Student’s *t*-test was conducted to evaluate the differences between control and experimental groups for RT-PCR. Values represent mean ± SD of at least six independent experiments. A Mann Whitney *U* test was conducted to evaluate the differences between data from control and experimental groups for Western blotting experiments. Values represent mean ± SD of at least three independent experiments. (^∗^*p* < 0.05 and ^∗∗^*p* < 0.01 for statistically significant differences).

## Data Availability Statement

All datasets generated in this study are included in the article/Supplementary Material.

## Ethics Statement

This study was carried out in accordance with the recommendations of Spanish guidelines (53/2013) of the Ethics Committee for Animal Experimentation (EAEC) of Principe Felipe Research Centre. The protocol was approved by the Ethics Committee for Animal Experimentation (EAEC) of Principe Felipe Research Centre.

## Author Contributions

FR-J conceived the idea, designed the research studies, conducted the experiments, acquired the data, analyzed the data, and wrote the manuscript. EC conducted the experiments and acquired the data. VM-M provided the conceptual advice. SE conceived the idea, provided the conceptual advice, designed the research studies, wrote the manuscript, and contributed to the materials.

## Conflict of Interest

The authors declare that the research was conducted in the absence of any commercial or financial relationships that could be construed as a potential conflict of interest.

## Supplementary Material

FIGURE S1 β-catenin/γ-tubulin and acetylated tubulin/γ-tubulin distribution in GFAP-TK mouse spinal cord-derived neurospheres cultured *in vitro*. GFAP-TK neurospheres were treated with Vehicle [DMSO (V)], 5-aza-dc, or GCV. **(A)** The β-catenin (green) antibody was used to mark cell borders. The γ-tubulin (red) antibody was used to detect single or groups of small basal bodies and two basal bodies. The identified pinwheel structures in each condition are marked with dashed lines for the periphery of ependymal cells and with continuous lines for the core formed by small central cells (schematic). Numbers mark the consecutive cores of adjacent pinwheels aligned horizontally (d). Asterisks were used to mark aberrant accumulations of γ-tubulin-positive small cells in the core and dots for aberrant accumulations of γ-tubulin-positive puncta within larger cells (schematic). **(B)** The acetylated tubulin (green) antibody was used to mark cilia. The γ-tubulin (red) antibody was used to detect single or groups of small basal bodies and two basal bodies. The identified pinwheel structures in each condition are marked with continuous lines for the core (schematic). Dotted arrows indicate the horizontal and oblique distribution of aligned cores in the vehicle (schematic). Asterisks were used to mark accumulations of acetylated tubulin/γ-tubulin-positive cells in the core and dots for aberrant accumulations of unstructured acetylated tubulin/γ-tubulin-positive puncta within cells (schematic). Scale bars, 20 μM.
