# Supplementary figures and images for: Organized Neurogenic-Niche-Like Pinwheel Structures Discovered in Spinal Cord Tissue-Derived Neurospheres
Source: Front Cell Dev Biol. 2019 Dec 20;7:334. doi: 10.3389/fcell.2019.00334 (PMC6932972; doi:10.3389/fcell.2019.00334)

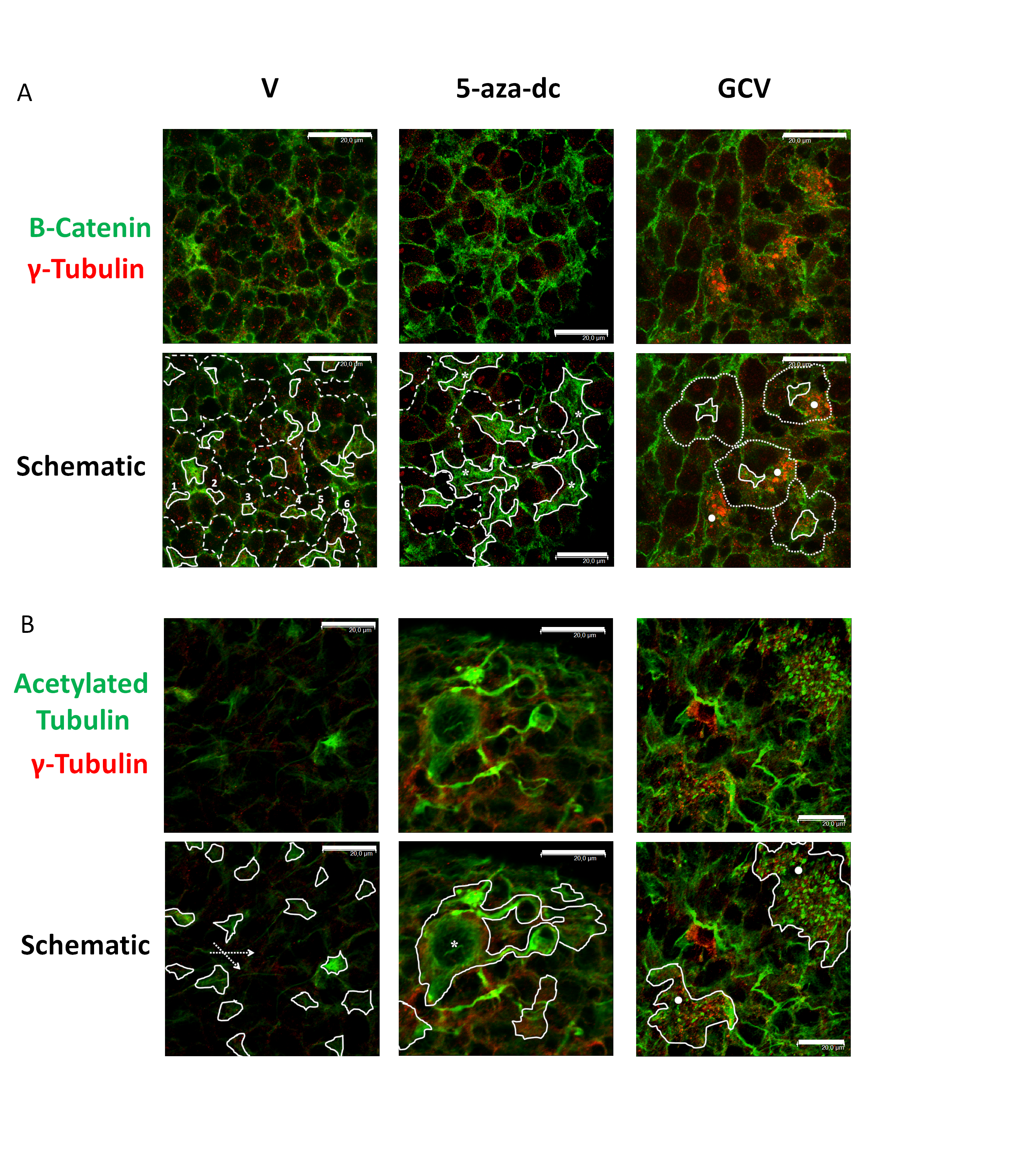

Supplement: FIGURE S1 — β-catenin/γ-tubulin and acetylated tubulin/γ-tubulin distribution in GFAP-TK mouse spinal cord-derived neurospheres cultured in vitro. GFAP-TK neurospheres were treated with Vehicle [DMSO (V)], 5-aza-dc, or GCV. (A) The β-catenin (green) antibody was used to mark cell borders. The γ-tubulin (red) antibody was used to detect single or groups of small basal bodies and two basal bodies. The identified pinwheel structures in each condition are marked with dashed lines for the periphery of ependymal cells and with continuous lines for the core formed by small central cells (schematic). Numbers mark the consecutive cores of adjacent pinwheels aligned horizontally (d). Asterisks were used to mark aberrant accumulations of γ-tubulin-positive small cells in the core and dots for aberrant accumulations of γ-tubulin-positive puncta within larger cells (schematic). (B) The acetylated tubulin (green) antibody was used to mark cilia. The γ-tubulin (red) antibody was used to detect single or groups of small basal bodies and two basal bodies. The identified pinwheel structures in each condition are marked with continuous lines for the core (schematic). Dotted arrows indicate the horizontal and oblique distribution of aligned cores in the vehicle (schematic). Asterisks were used to mark accumulations of acetylated tubulin/γ-tubulin-positive cells in the core and dots for aberrant accumulations of unstructured acetylated tubulin/γ-tubulin-positive puncta within cells (schematic). Scale bars, 20 μM. [file Image_1.TIF]
